# Definition, Incidence, Prediction, and Prevention of Bleeding Events After Transcatheter Aortic Valve Implantation

**DOI:** 10.3390/jcm14207154

**Published:** 2025-10-10

**Authors:** Iosif Xenogiannis, Ioannis Lianos, Grigoris V. Karamasis, Charalampos Varlamos, Fotios Kolokathis, Christos Pappas, Stamatia Kovra, Konstantinos Tsaousidis, Christos Mourmouris, Antonis N. Pavlidis, Andreas S. Triantafyllis, Andreas S. Kalogeropoulos

**Affiliations:** 1 Department of Cardiology, Mitera General Hospital, Hygeia HealthCare Group, 15123 Athens, Greece; ixenogiannis@mitera.gr (I.X.); lianosyi@gmail.com (I.L.); skovra@mitera.gr (S.K.); cmourmouris@hygeia.gr (C.M.); 2 Second Department of Cardiology, Attikon University Hospital, National and Kapodistrian University of Athens, 12462 Athens, Greece; grigoris.karamasis@gmail.com (G.V.K.); chvar@yahoo.com (C.V.); fotisks@hotmail.com (F.K.); clincard@hotmail.com (C.P.); 3 Department of Cardiology, St Thomas’ Hospital, Guy’s and St Thomas’ NHS Foundation Trust, London SE1 9RT, UK; antonis.pavlidis@gstt.nhs.uk; 4 Abbott, 17456 Athens, Greece; 5 Department of Cardiology, Asklepion General Hospital, 11528 Athens, Greece; andtridoc@yahoo.gr

**Keywords:** transcatheter aortic valve implantation (TAVI), bleeding, ultrasound guidance, antiplatelets, femoral access, computed tomography, anticoagulation

## Abstract

Bleeding remains the most common complication following transcatheter aortic valve implantation (TAVI), despite a decline in its incidence over time. Periprocedural (≤30 days) major or life-threatening bleeding is reported to occur in 2.0–6.6% of patients undergoing TAVI. Major bleeding events carry a significant risk of mortality, with rates of 14.1% at 30 days and 27.8% at one year. The timely identification and management of patients at an elevated risk are therefore essential. Preventive measures include optimizing antithrombotic therapies, utilizing ultrasound-guided femoral access, employing single arterial access or a radial artery for secondary access, and administering unfractionated heparin under close monitoring. Long-term follow-up is essential for recognizing and managing late hemorrhages. In this review, we aimed to provide an in-depth analysis of bleeding events associated with TAVI and the most recent updates regarding the antithrombotic therapy of TAVI patients and its clinical impact.

## 1. Introduction

Transcatheter aortic valve implantation (TAVI) for symptomatic patients with severe aortic stenosis is indisputably one of the foremost advancements in interventional cardiology in the 21st century.

Candidates undergoing TAVI tend to have an elevated risk of bleeding complications, as these patients are typically elderly, frail, and present with multiple comorbidities. In addition, TAVI necessitates the use of larger arterial sheaths inserted via the transfemoral approach. In contrast to percutaneous coronary interventions (PCIs), where the radial artery is frequently selected due to its association with a lower mortality and reduced bleeding rates, the radial artery is generally not feasible as the primary access site for TAVI [1]. Although significant advances in the technology and techniques of TAVI—such as the use of smaller and more hydrophilic slippery sheaths, the widespread adoption of ultrasound-guided femoral access, and the selection of lower-risk patients—have been adapted over the last few years, a substantial percentage of patients develop clinically relevant bleeding after the procedure. In the early era of TAVI, it was estimated that 14.7–46.2% of patients experienced major, life-threatening, or disabling bleeding at one year after the procedure. More recently, this rate has decreased to 3.2–13.1%, which, although significantly lower, remains noteworthy [1,2,3,4,5,6,7,8,9]. Importantly, major bleeding has been consistently associated with an increased rate of mortality [10,11]. Therefore, the identification of vulnerable patients and the subsequent application of preventive measures are of utmost importance.

In this paper, we provide an overview of the definitions and current classifications for bleeding in patients undergoing TAVI. Additionally, we examine the factors associated with bleeding and discuss preventive measures that may reduce the risk.

## 2. Bleeding Definitions, Incidence, and Current Classifications

Bleeding remains the most common complication after TAVI [12]. It is generally classified as early (<30 days from the procedure) or late (>30 days from the procedure) [12].

In the vast majority of cases, early bleeding is caused by periprocedural complications. According to the results from the large CENTER (cerebrovascular events in patients undergoing transcatheter aortic valve Implantation with balloon-expandable valves versus self-expandable valves) study, which included 23,562 patients who underwent transfemoral TAVI, the 30-day major bleeding rate was 6.6% and was caused mainly by vascular-access-related bleeding (70%). Subsequent cases involved pericardial bleeding (13.5%), gastrointestinal bleeding (5.1%), and urogenital bleeding (3%) [10]. The results from the previous study are in accordance with reports from national TAVI registries, which estimate that 3.3–13.1% will develop a major bleeding event within the first 30 days post-TAVI [6,7,8,9]. Greater operator experience, the use of smaller 14–18 Fr sheaths, ultrasound-guided femoral artery access, and expanding TAVI to lower-risk patients have all reduced the frequency of bleeding events over time [9,10].

Late bleeding events remain relatively frequent in patients who have undergone TAVI. It is estimated that 5.2–11.3% will develop a major late bleeding event [11,13,14]. Similarly to early bleeding events, late bleeding events are associated with an increased long-term mortality [11,14]. The most frequent site of late bleeding is gastrointestinal (roughly 41–43%), followed by neurological (7.1–25.7%) and genitourinary (6.3–14.3%) bleeding [11,13,14].

The Bleeding Academic Research Consortium (BARC) classification has been used as the standard classification for defining and categorizing bleeding events in clinical trials [15]. The Valve Academic Research Consortium (VARC) was founded in 2010, with the intention to identify appropriate clinical endpoints and standardize the definitions of these endpoints, specifically for transcatheter and surgical aortic valve clinical trials [16]. The rapid evolution of TAVI and its widespread application worldwide has led the VARC group to recently update the standardized endpoint definitions for relevant clinical endpoints in TAVI. With respect to bleeding events, VARC-3, in accordance with the BARC classification, uses a more detailed and comprehensive description for the definition of bleeding events: type 1 (minor; type BARC 2 and 3a), type 2 (major; type BARC 3a), type 3 (life-threatening; type BARC 3b, 3c, and 4), and type 4 (leading to death, either probable or definite; type BARC 5a and 5b, respectively) [17].

In Table 1, the primary factors associated with both early and late post-TAVI bleeding are presented.

## 3. Patient-Related Factors

A variety of patient-related factors are associated with the appearance of bleeding post-TAVI.

The female gender has been reported as a risk factor for bleeding in multiple studies [18,19,20]. In a study by Picolo et al., the female gender was related with life-threatening or major access-site-related bleeding (hazard ratio [HR]: 2.59%; 95% confidence interval [CI]: 1.1–6.13) [18]. Vlastra et al. sought to examine the differences between women and men undergoing TAVI using data from the large CENTER database. Women had a 50% higher chance of experiencing life-threatening or major bleeding [20]. Finally, in a large cohort study from the US, the female gender was an independent factor associated with in-hospital bleeding (HR: 1.53; 95% CI: 1.39–1.68) [19].

Chronic kidney disease (CKD) has been identified as a predictor of post-procedural bleeding. According to Li et al., patients with CKD—defined as an estimated glomerular filtration rate (eGFR) below 60 mL/min/1.73 m^2^—showed a higher incidence of bleeding one year after TAVI (9.2% compared to 4.9%, *p* = 0.032). Patients with an eGFR < 30 mL/min/1.73 m^2^ had bleeding rates up to 16.5%, showing that the bleeding risk rises as CKD advances. Similarly, data from the National Inpatient Sample (NIS) database indicate that CKD is independently associated with a higher risk of in-hospital major bleeding (odds ratio [OR]: 1.35; 95% CI: 1.27–1.44) [21]. Similarly, according to a report derived from the National Inpatient Sample (NIS) database, CKD is independently associated with an increased risk of in-hospital major bleeding (odds ratio [OR]: 1.35; 95% CI: 1.27–1.44) [22].

Peripheral artery disease (PAD) is common among patients undergoing TAVI, with the prevalence rates ranging from 4.1% to 43% [4,5]. Previous studies indicate that PAD patients have a higher likelihood of in-hospital (OR: 1.37; 95% CI: 1.25–1.50) and one-year (HR: 1.18; 95% CI: 1.09–1.27) bleeding events [19,23]. NIS data indicate that PAD patients undergoing TAVI had higher transfusion rates for a hemorrhage than those without PAD (14.1% vs. 11.7%, *p* < 0.001) [24]. Bansal et al. found that PAD patients receiving a peripheral intervention alongside TAVI were more likely to need blood transfusions than those undergoing TAVI alone (16.0% vs. 11.3%, *p* < 0.001) [25].

Aortic stenosis can induce the development of type 2A von Willebrand disease. With respect to the pathophysiology of the disease, the passage of the von Willebrand factor (vWF) through a stenotic valve can lead to its cleavage to inactive fragments that results in suboptimal hemostasis. Interestingly, TAVI has been shown to normalize vWF, reducing bleeding diathesis [24]. Significant paravalvular leaks can result in sustained high shear stress, which predisposes patients to von Willebrand factor malfunction and increases the risk of late bleeding following TAVI [13,14].

Heyde’s syndrome consists of the co-occurrence of aortic stenosis and gastrointestinal bleeding caused by angiodysplasias. It is estimated that it affects 6.3% of patients who undergo TAVI, and it is recognized as a periprocedural bleeding risk factor [26]. In the same line with type 2A vWF disease, the correction of aortic stenosis via TAVI resolves gastrointestinal bleeding in the vast majority of patients with Heyde’s syndrome, while paravalvular leaking mitigates its positive effect [26].

In a study conducted by Dvir et al., significant thrombocytopenia (platelet count below 100 × 10^9^/L) was observed in 36.1% of patients, typically occurring within two days post-TAVI. Those with severe thrombocytopenia (platelet count below 50 × 10^9^/L) faced notably higher rates of major or life-threatening bleeding, as well as increased 30-day mortality rates [27].

## 4. Antithrombotic Therapy

Antithrombotic therapy following TAVI is essential to prevent thromboembolic complications, such as a stroke or valve thrombosis. The optimal antithrombotic scheme in such patients has evolved and continues to be refined based on recent trial data and updated guidelines. The small ARTE (aspirin versus aspirin + clopidogrel following transcatheter aortic valve implantation) randomized controlled trial (RCT) showed that single antiplatelet therapy (SAPT) reduced the risk of major or life-threatening events without increasing the risk of a myocardial infarction or stroke compared with dual antiplatelet therapy (DAPT) (15.1% vs. 26.6%, *p* = 0.001) [28]. In accordance with the previous RCT, Brouwer et al. reported that, in patients after TAVI with no indication for long-term oral anticoagulation therapy (OAC), the use of SAPT with aspirin reduced the rate of bleeding and non-procedural bleeding compared to three-month DAPT with clopidogrel and aspirin (15.1% vs. 24.9%, *p* = 0.005) [29]. The rate of the composite endpoint of death from cardiovascular causes, non-procedure-related bleeding, a stroke, or a myocardial infarction was lower in the aspirin monotherapy group (23% vs. 31.1%, *p* < 0.001), while there was no difference in the rate of thromboembolic events [29]. The previous results were recently confirmed by a real-world data analysis from the multicenter TRITAVI (Transfusion Requirements in Transcatheter Aortic Valve Implantation) registry, which included 5514 patients who underwent TAVI among 20 European centers. At 6 months, the SAPT group had fewer bleeding events (0.5% vs. 1.3%, *p* = 0.001) and showed a trend towards fewer major ischemic events (0.4% vs. 0.7%, *p* = 0.07). Notably, the overall mortality was about one-third lower with SAPT, with significant reductions in both cardiovascular and non-cardiovascular deaths [30]. In the GALILEO (global study comparing a rivaroxaban-based antithrombotic strategy to an antiplatelet-based strategy after transcatheter aortic valve replacement to optimize clinical outcomes) RCT, the co-administration of daily low-dose rivaroxaban (10 mg) plus low-dose aspirin (75–100 mg daily) was associated with a higher risk of death or thrombo-embolic complications and a higher risk of bleeding compared with an antiplatelet-based treatment consisting of low-dose aspirin plus clopidogrel (75 mg daily), leading to the premature termination of the trial [31].

In addition, a substantial percentage of patients who undergo TAVI have concomitant coronary artery disease and/or an indication for receiving therapeutic anticoagulation therapy. It is estimated that approximately 40% of TAVI candidates suffer from concomitant atrial fibrillation [32]. This complex patient population has also been the focus of research in recent years regarding the most effective and safe antithrombotic therapy.

In the ENVISAGE TAVI AF trial, TAVI patients with atrial fibrillation were randomized to receive either edoxaban or vitamin K antagonists [33]. Although edoxaban was non-inferior to vitamin K antagonists with respect to the composite primary outcome of adverse clinical events, the administration of edoxaban was related to a higher rate of major bleeding events (9.7% vs. 7%), driven mainly by major gastrointestinal bleeding (5.4% vs. 2.7%). It is worth noting that approximately 60% of the patients in both arms were on an oral antiplatelet therapy after TAVI. Furthermore, regarding the patients who received specified concomitant antiplatelet therapy, the use of edoxaban led to a higher percentage of major bleeding compared to those who received vitamin K antagonists. In the POPular-TAVI RCT cohort B trial, three months of clopidogrel added to oral anticoagulation increased serious bleeding at one month and one year in eligible patients [34]. The ATLANTIS (anti-thrombotic strategy to lower all cardiovascular and neurologic ischemic and hemorrhagic events after transaortic valve implantation for aortic stenosis) trial investigated apixaban vs. the standard of care in the clinical setting of TAVI [32]. The standard-of-care group was further stratified based on patients’ preexisting need for OAC. In stratum 1, where OAC was indicated, apixaban was compared with vitamin K antagonists, while in stratum 2, where there was no indication for OAC, apixaban was compared with antiplatelet therapy (DAPT was administered in 56.9% of the patients). No differences between the two arms were noticed regarding the primary endpoint of thrombotic or bleeding events. On the other hand, apixaban was found to reduce the incidence of obstructive valve thrombosis in patients that did not have an existing reason to be on OAC. However, it is crucial to note that, while apixaban reduced this specific type of valve thrombosis, this finding did not translate into a reduction in the overall rate of clinical events like death, stroke, or major bleeding. In fact, in this same subgroup, there was a signal of a higher non-cardiovascular mortality with apixaban compared to antiplatelet therapy alone. The most recent European guidelines, based on the previous RCTs, recommend lifelong OAC for TAVI patients with an OAC indication, and 12 months of SAPT for those without (class I, level B and class I, level A evidence, respectively) [35]. Table 2 summarizes the key trials on the optimal antithrombotic therapy in TAVI patients.

The continuation or interruption of OAC before TAVI is a matter of ongoing debate. Whilst metanalyses of observational studies have indicated that continuing OAC may be associated with a reduced incidence of a stroke without an increased risk of bleeding, these findings were not supported by the only existing randomized controlled trial to date [36,37,38]. The POPular PAUSE TAVI (periprocedural continuation versus interruption of oral anticoagulant drugs during transcatheter aortic valve implantation) randomized patients with an indication for OAC undergoing TAVI to either stop OAC according to the recommendations of the current guidelines for patients who undergo high-bleeding-risk surgery or continue OAC without interruption (18% of the patients received vitamin K antagonists, whilst the rest received a direct oral anticoagulant). The study showed that continuing oral anticoagulation (OAC) was noninferior to interruption regarding the composite primary endpoint, which included cardiovascular death, strokes, myocardial infarctions, major vascular complications, or major bleeding within 30 days. However, the continuation group did not meet the non-inferiority margin for overall and procedure-related bleeding events. These results indicate that the periprocedural discontinuation of OAC is a clinically acceptable approach [38].

## 5. Procedure-Related Factors

Even though the femoral artery is considered the gold standard for access in TAVI interventions, in a substantial percentage of patients (estimated at up to 10%), femoral access is not feasible [39]. Multiple alternative access sites have been used, such as transapical, transaortic, and transcarotid sites, while more recently, transaxillary/subclavian and transcaval access have gained popularity due to their less invasive nature. Historically, major bleeding has been found to be more frequent with the use of transapical or subclavian approaches compared with the transfemoral approach, although, in experienced operator hands and after adjusting for confounding factors, the percentage of major or life-threatening bleeding might be similar between the transapical and transfemoral approaches [40,41]. Modern alternative approaches, as described above, seem to have overcome this drawback of older non-femoral approaches. In a recent cohort study, major or life-threatening bleeding was significantly lower with the use of transcarotid vs. transapical or transaortic approaches (4.3% vs. 19.9%; *p* = 0.002) [42]. A large metanalysis that included 3992 patients using propensity score-matching reported no difference in terms of bleeding (OR, 1.05; 95% CI, 0.68–1.61) between transfemoral and transcarotid/subclavian access sites [43].

In the early era of TAVI, sheath caliber delivery systems ranged between 18 and 22 Fr. The reduction in sheath sizes with respect to the second- and third-generation TAVI delivery systems to 14–18 Fr was accompanied by a substantial reduction in vascular complications, including bleeding [44]. According to a report from the Society of Thoracic Surgeons/American College of Cardiology Transcatheter Valve Therapies Registry, bleeding events related to TAVI operations have declined over time. Furthermore, investigators have reported that a sheath size > 17 Fr (OR, 1.38; 95% CI: 1.25–1.53) and femoral cutdown (OR, 1.28; 95% CI: 1.13–1.46) were correlated with a higher incidence of in-hospital bleeding events [19]. In a study by Van Kesteren et al., the sheath-to-iliofemoral-artery ratio predicted major vascular complications after TAVI (unadjusted OR, 7.51; 95% CI: 1.61–34.95). In the same study, the occurrence of vascular complications was correlated with statistically significantly higher rates of life-threatening or major and minor bleeding events compared with patients who experienced no vascular complications (27.7% and 55.4% vs. 4.4% and 0.6%, respectively, *p* ≤ 0.001) [45]. Likewise, Languet et al. reported that major vascular complications were associated with higher rates of major/life threatening bleeding compared with minor or no vascular complications (42.8% vs. 0% vs. 0.6%, respectively, *p* = 0.001) [46]. The usage of the radial artery for secondary access during TAVI has led to a lower incidence of major vascular (1.8% vs. 0%, *p* < 0.001) and major/life-threatening (1% vs. 0%, *p* < 0.001) complications [47]. In a retrospective analysis, including 100 consecutive patients undergoing TAVI with SAPIEN 3 and SAPIEN 3 Ultra bioprostheses, evaluating the feasibility and safety of a single-access transfemoral technique for the entire TAVI procedure (no secondary arterial access or venous access) found significantly lower rates of major vascular and bleeding complications compared to a dual arterial access approach [48].

Another key procedural aspect linked to bleeding complications after TAVI is the technique used for large-bore vascular hemostasis following the procedure. In this respect, two recent randomized trials have evaluated the superiority and efficacy of a more contemporary hybrid strategy using a suture-based approach (single ProGlide) in combination with a plug-based approach (Angioseal) over the conventional suture-based approach with two ProGlides [49,50]. In both studies, the hybrid approach was associated with significantly fewer access-site-related vascular complications and life-threatening or clinically significant bleeding. Table 3 presents the principal clinical trials that have evaluated large-bore closure techniques and their associated outcomes.

In recent years, the adoption of ultrasound-guided arterial puncture has been widely adopted over conventional fluoroscopic guidance to gain femoral artery access during TAVI. An overwhelming body of evidence from clinical studies, including large-scale registries and meta-analyses, has firmly established that using ultrasound guidance for femoral artery puncture significantly reduces bleeding and vascular complications during TAVI. This technique has become a cornerstone of best practices for achieving safe transfemoral access in this high-risk patient population.

A comprehensive meta-analysis analyzed data from eight observational studies involving over 3800 patients. The findings were compelling in favor of ultrasound guidance, as the latter was associated with a 50% reduction in major and minor access site vascular complications. In addition, there was a significant reduction in total access site bleeding complications [54].

The PULSE registry, a large real-world multicenter registry, conducted a propensity-matched analysis comparing ultrasound-guided punctures to the traditional fluoroscopy-guided approach. The results demonstrated that the ultrasound-guided group had lower rates of the primary endpoint, which was a composite of access-related vascular complications and significant bleeding, corresponding to a notable reduction in bleeding events (type II–IV) [55].

## 6. Scores

Two bleeding risk models that aspire to predict the risk of bleeding after TAVI have been developed: the more practical PREDICT-TAVR and the model recently proposed by the Valve Academic Research Consortium for High Bleeding Risk (VARC-HBR) [44,56].

The first consists of a six-item score (blood hemoglobin, serum iron, creatinine clearance, common femoral artery diameter, dual antiplatelet therapy, and anticoagulant therapy). According to the total score collected, patients are separated into bleeding risk quartiles: low risk, ≤8; moderate risk, >8 and ≤10; high risk, >10 and ≤12; and very high risk, >12. The model demonstrated a high discriminative ability to predict bleeding events at 30 days, with the 30-day area under the receiver-operating characteristic curve (AUC) being 0.80 (95% confidence interval [CI]: 0.75–0.83); however, it failed to predict bleeding events between 30 days and 1 year [56].

The very recent model proposed by VARC-HBR includes 15 major and 6 minor bleeding criteria. The patients are dissected into three categories: patients with only one minor criterion are considered as moderate-risk, with an estimated incidence of 1-year BARC type 3–5 bleeding < 4%; patients with one major or two minor criteria are considered as high-risk, with an estimated risk for 1-year BARC type 3–5 bleeding ≥ 4% to <8%; and finally, patients with at least two major or three minor criteria are characterized as very-high-risk patients for bleeding, with an estimated incidence of 1-year BARC type 3–5 bleeding ≥ 8% [44]. The model was validated in a large multicenter study that included 10,449 patients undergoing TAVI [57]. Approximately nine in ten patients had at least one VARC-HBR criterion. The most met major and minor criteria were anemia (23.9%) and DAPT (31.9%), respectively. Patients that were classified as moderate-risk according to the VARC-HBR bleeding risk stratification had a higher frequency of one-year BARC 3 or 5 bleeding events compared with high- and very-high-risk patients (8.8% vs. 12.3% vs. 17.5%, respectively, *p* < 0.001). Of note, the observed rates of hemorrhagic events were higher than the predicted rates proposed by the VARC-HBR consensus [57]. Finally, the one-year mortality was higher for the high- and very-high-risk patients (HR, 1.33 [95% CI, 1.04–1.70] and 1.97 [95% CI, 1.53–2.53], respectively) compared with the low-risk patients who met none of the VARC-HBR criteria [57].

## 7. Preventive Measures

Careful medical history recording, a meticulous physical examination, and a review of basic lab tests (such as the hemoglobulin, platelet count, INR, a-PTT, and creatinine) are the initial steps for the recognition of a patient with a high risk of bleeding. Ideally, the use of the above-mentioned bleeding risk scores will give a more objective picture about the risk for hemorrhagic events by quantifying it and predicting specific percentages for bleeding complications. The identification and correction of anemia and thrombocytopenia is crucial for the avoidance of future bleeding after TAVI. Furthermore, sites of potential blood loss in patients with unexplained anemia should be examined, i.e., with the performance of a gastroscopy/colonoscopy and a cystoscopy in the case of a positive urine blood test, and the underlying causes of bleeding should be treated if feasible (i.e., the administration of proton pump inhibitors in the case of a peptic ulcer, or a polypectomy for colon or bladder polyps). A nephrology consultation for patients with CKD before the procedure is paramount. The avoidance/interruption of any nephrotoxic agents, the optimization of fluid status, and epoetin administration in cases of CKD-related anemia may prevent the deterioration of renal function and unnecessary blood transfusions post-TAVI.

Every patient who undergoes TAVI should be on the appropriate antithrombotic regimen before the operation. SAPT (low-dose aspirin) is the default option for patients who have not undergone a stent implantation in the last 6 or 12 months before TAVI for a chronic coronary syndrome (CCS) or an acute coronary syndrome (ACS), respectively. It is estimated that roughly 20% of patients who have TAVI undergo a PCI shortly before, during, or after the operation [58]. The new stent technology has allowed, according to the most recent European guidelines, the shortening of DAPT to one month post-PCI for high-bleeding-risk patients, irrespective of the clinical setting (CCS or ACS) [59,60]. Although there are no available data, according to a consensus document of the ESC Working Group on Thrombosis and the European Association of Percutaneous Cardiovascular Interventions (EAPCI), in collaboration with the ESC Council on Valvular Heart Disease, DAPT (or NOAC plus SAPT for patients with an indication for NOAC) is recommended to be continued for patients who have undergone a PCI within three months prior to TAVI. According to the same document, the further continuation of DAPT post-TAVI for up to six months should be tailored according to the clinical setting (CCS vs. ACS) and patient profile (high-bleeding vs. high-thrombotic risk) [58]. NOAC or vitamin K antagonists should be used as monotherapy when indicated without antiplatelet co-administration in patients with no recent PCI. According to the outcomes of the POPular PAUSE TAVI, it is reasonable to discontinue NOAC 48 h (or earlier in patients with renal failure), acenocoumarol therapy 72 h, and phenprocoumon or warfarin therapy 120 h before TAVI.

In addition, the widespread adoption of pre-procedural computed tomography (CT) angiography has been a transformative development in transcatheter TAVI, playing a pivotal role in the dramatic reduction in procedural complications, most notably bleeding and vascular events. Once considered an adjunct, a comprehensive pre-procedural CT scan is now the undisputed standard of care, providing an essential roadmap for the heart team to plan and execute the procedure with an enhanced safety and precision.

**Figure 1:**
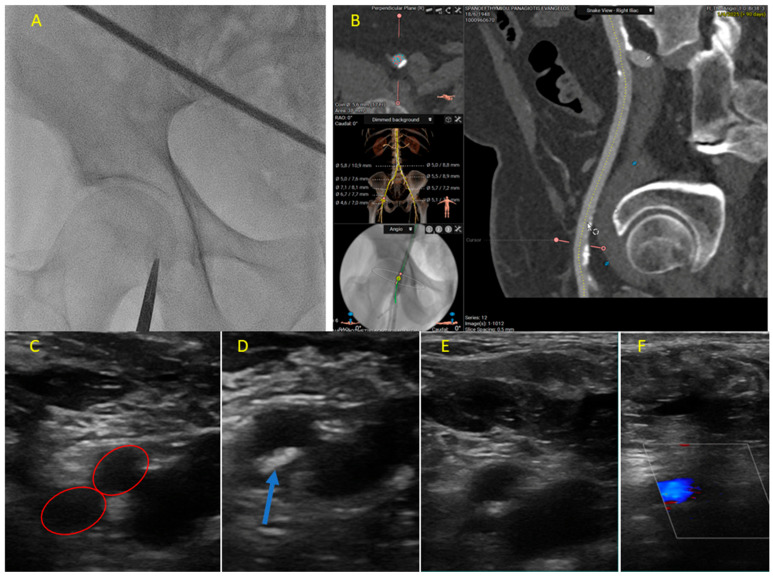
Multimodality imaging for femoral access puncture in patients undergoing transcatheter aortic valve implantation. (**A**) Detection of the head of the femoral bone via fluoroscopy. Forceps can be used to easily indicate the middle segment of the femoral head as the ideal point for puncture at the middle third of the common femoral artery. (**B**) Co-registration of the fluoroscopy image with the CT angiography. A calcified atherosclerotic segment of the common femoral artery was detected by CT angiography (red lines) and projected over the fluoroscopy image (yellow circle). In addition, the site of the bifurcation between the profunda and the superficial femoral artery is noted below the middle third of the femoral head. Finally, we can clearly identify the optimal site for arterial puncture just at the beginning of the atherosclerotic plaque, corresponding to just above the mid-level of the femoral head. (**C**) The point of bifurcation into the superficial femoral artery and profunda (red circles) was also identified by ultrasound. (**D**) The area of calcification and atherosclerotic plaque was recognized (blue arrow), and the probe was moved higher based on the initial mark made by the forceps to identify the middle third of the femoral head. (**E**) The ideal point for puncture above the area of calcified atherosclerotic plaque and at the healthiest segment of the vessel before transition to the external iliac artery was selected. (**F**) The presence of brisk flow in the common femoral artery was confirmed by color Doppler after final closure (white rectangle).

The primary mechanism by which CT angiography mitigates the bleeding risk is through its detailed and comprehensive assessment of the patient’s unique anatomy, from the aortic valve down to the femoral arteries. This allows for a proactive strategy to avoid potential hazards that were previously encountered with less certainty during the procedure. The key contributions of CT angiography to bleeding reduction involve the following:

**Precise Vascular Access Planning:** Most bleeding complications in TAVI are related to the arterial access site in the groin. CT angiography provides precise, sub-millimeter measurements of the entire iliofemoral arterial system. This detailed mapping allows clinicians to measure the exact vessel diameter and ensure that the femoral and iliac arteries are large enough to accommodate the TAVI delivery sheath, preventing arterial dissection or rupture. Calcification should be identified and location and severity of calcified plaques should be visualized. Puncturing through a heavily calcified plaque can lead to vessel trauma and prevent the effective use of vascular closure devices, causing severe bleeding. The tortuosity of the iliac arteries should be assessed. Navigating highly tortuous vessels with a large sheath can lead to vascular injury. The optimal puncture site should be optimized by providing a clear 3D picture; CT, often used in conjunction with fluoroscopy and ultrasound guidance during the procedure, ensures that the needle entry into the femoral artery is in an optimal, disease-free location (Figure 1).

By identifying a challenging or prohibitive iliofemoral anatomy beforehand, clinicians can opt for alternative access routes (e.g., subclavian, transcarotid, or transapical) that are safer for that specific patient, thereby avoiding a high-risk femoral attempt that could lead to a life-threatening hemorrhage.

**Accurate aortic annulus sizing:** While not directly related to access site bleeding, the correct sizing of the aortic annulus is critical to overall procedural success and avoiding other catastrophic bleeding events. CT allows for precise, multiplanar measurements of the aortic valve annulus, ensuring that the correct size of the TAVI prosthesis is selected and avoiding deploying an oversized or undersized valve, thus preventing either an annular rupture or a significant paravalvular leak, respectively.

During the TAVI procedure, the femoral artery should be the access site of choice, followed by transcarotid, transaxillary/subclavian, and transcaval access, preferably in centers with substantial experience in gaining access through alternative access sites, with transaortic and transapical access being the least preferable options. The radial artery is highly recommended as the default secondary arterial access, whereas in centers with significant experience and with the use of balloon-expandable valves, a single arterial access for the entire TAVI procedure seems to be a favorable strategy for preventing bleeding complications.

In addition, an ultrasound-guided femoral puncture is highly recommended to minimize vascular and subsequent bleeding complications. In the event that there is a lack of randomized data, multiple retrospective studies have shown that the utilization of ultrasound for gaining access has reduced vascular complications and bleeding events in TAVI procedures; therefore, this should be used as the standard of practice [54,55,61,62]. Ultrasound allows the operator to recognize the point of femoral bifurcation and guide the femoral puncture above it, while at the same time avoiding severely diseased/calcified segments [63]. The traditional method of femoral artery access relied on anatomical landmarks and fluoroscopy (X-ray guidance), which often resulted in suboptimal needle placement. Punctures that are too high, are too low, or hit a calcified plaque can lead to a host of complications, including severe bleeding, pseudoaneurysms, and arterial dissection. Ultrasound guidance addresses these challenges by providing the real-time visualization of the vascular anatomy. In this context, a previous prospective analysis of 1022 consecutive patients undergoing transfemoral TAVI with a less invasive totally endovascular (LITE) technique, involving an ultrasound-guided femoral artery puncture and guidewire insertion, a radial or ulnar approach for secondary arterial access, and non-invasive pacing (by ventricular guidewire or a pacemaker external programmer), showed notably low rates of access-related major vascular complications and VARC-3 ≥ type 2 bleedings (1.2%) [64].

During the procedure, unfractionated heparin is the preferred anticoagulant, with bivalirudin used as an alternative in patients with a history of heparin-induced thrombocytopenia, since it has shown no benefit over heparin in terms of major bleeding or adverse cardiovascular events [65]. Meticulous anticoagulation status monitoring during the operation is mandatory, targeting an ACT of 250–300 sec or higher [58,66]. Protamine sulphate administration results in significantly lower rates of life-threatening and major bleeding complications without increasing thrombotic events; thus, it is recommended at the end of the procedure [67]. The exact time of protamine administration remains unclear, with some operators preferring to give protamine before vascular closure while others opt to administer protamine after vascular closure [68]. The full antagonization of heparin has been associated with lower rates of life-threatening and major bleeding after TAVI compared to partial heparin reversal [69]. Data regarding the safest closure device in terms of reducing bleeding events between plug-based (MANTA, Teleflex, Wayne, Pennsylvania, USA) and suture-based (ProGlide, Abbott Vascular, Illinois, USA) devices are conflicting [51,52]. It is likely that ultrasound-guided MANTA deployment reduces hemorrhagic events compared with no ultrasound utilization [70,71]. In addition, a hybrid approach using combined suture-based and plug-based hemostasis has demonstrated superior outcomes with reduced vascular complications and bleeding events compared to a suture-only-based strategy with two ProGlides, and it is highly advocated [49,50].

A paravalvular leak is a strong predictor for major/life-threatening complications; thus, every effort to prevent it should be taken [13].

**Figure 2:**
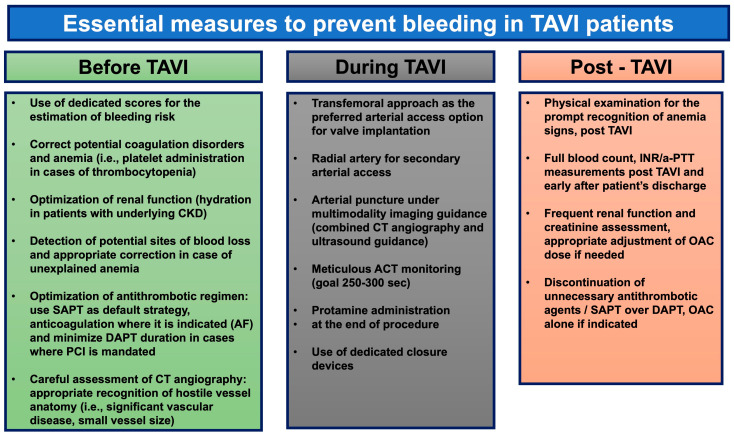
Essential measures for bleeding prevention in patients who undergo TAVI. ACT: activated clotting time; DAPT: dual antiplatelet therapy; OAC: oral anticoagulation therapy; SAPT: single antiplatelet therapy; TAVI: transcatheter aortic valve implantation.

The close monitoring of the patient based on (1) their history, with specific questions made about the presence of blood in their urine/stools, (2) a physical examination focused on the recognition of symptoms/signs of anemia, and (3) lab tests (full blood count, INR, a-PPT, and creatinine levels so as to adjust the dose or even change/halt anticoagulant agents based on the EGFR when indicated) should be continued after the completion of TAVI during the hospital stay and on subsequent follow-up visits for the prompt recognition and management of bleeding complications. The careful review of a patient’s medication is crucial in order to halt any unnecessary antithrombotic agents. The preventive measures are outlined in Figure 2.

## 8. Conclusions

Although less frequent than in the early years of TAVI, bleeding events remain among the most dreadful complications of the procedure. The recognition of high-risk patients is the first critical step, followed by the application of all the possible preventive measures to decrease the chances of a hemorrhage, such as a comprehensive CT TAVI vascular analysis, an ultrasound-guided femoral puncture, appropriate hemostasis with a hybrid (combined plug- and suture-based) strategy, radial secondary arterial access, guideline-based antithrombotic therapy, and the reversal of anticoagulation post-TAVI. Given the high rate of late bleeding events, patient monitoring for bleeding should not stop with their discharge from the hospital after a successful TAVI operation, but rather, it must continue—ideally indefinitely.

## Figures and Tables

**Table 1:** Risk factors for early and late bleeding complications following transcatheter aortic valve implantation (TAVI).

Category	Early Bleeding (≤30 Days)	Late Bleeding (>30 Days)
**Patient-Related Factors**	Advanced age (>80 years) Female sex Obesity (BMI ≥ 30 kg/m^2^) * Chronic kidney disease (eGFR < 30 mL/min) Anemia (Hb < 10 g/dL) Prior bleeding history Frailty	Advanced age (>80 years) Obesity (BMI ≥ 30 kg/m^2^) * Chronic kidney disease (eGFR < 30 mL/min) Anemia (Hb < 10 g/dL) Gastrointestinal angiodysplasia Liver disease Malignancy
**Procedure-Related Factors**	Large sheath size (>18 F) Vascular access complications Transfemoral approach Periprocedural stroke Valve malpositioning	Residual aortic regurgitation Device thrombosis (rare)
**Treatment-Related Factors**	DAPT Oral anticoagulation (e.g., high INR) Peri-procedural heparin Failure of vascular closure device	Oral anticoagulation DAPT Non-compliance with antithrombotic therapy Drug-drug interactions (e.g., NSAIDs)

* Obesity is associated with vascular complications and an altered drug metabolism. DAPT: dual antiplatelet therapy; BMI: body mass index; INR: international normalized ratio; NSAIDs: non-steroidal anti-inflammatory drugs.

**Table 2:** Randomized and major comparative trials on optimal antithrombotic therapy in patients undergoing TAVI.

Study (Year)	Population and Number of Patients	Strategies Compared	Primary Endpoint/Follow-Up	Key Outcomes
**ARTE (2017) [28]**	*n* = 222, no OAC	Aspirin vs. aspirin plus clopidogrel (3 months)	Major/life-threatening bleeding	Major life-threatening bleeding: 3.6% SAPT vs. 10.8% DAPT (*p* = 0.038); no difference in stroke/MI
**POPular-TAVI Cohort A (2020) [29]**	*n* = 665, no OAC	Aspirin alone vs. aspirin plus clopidogrel (3 months)	All bleeding events (VARC-2) at 12 months	Bleeding events: 15.1% SAPT vs. 26.6% DAPT (*p* = 0.001); CV death/MI/stroke: 23% vs. 31.1% (*p* < 0.001)
**POPular-TAVI Cohort B (2020) [34]**	*n* = 313, OAC indication	OAC alone vs. OAC plus clopidogrel (3 months)	All bleeding events (VARC-2) at 12 months	Bleeding events: 21.7% OAC alone vs. 34.6% OAC plus clopidogrel (*p* = 0.01); no ischemic benefit from adding clopidogrel
**GALILEO (2020) [31]**	*n* = 1644, no OAC	Rivaroxaban 10 mg plus aspirin vs. aspirin plus clopidogrel	Composite of death/thromboembolism/bleeding and life-threatening, disabling, or major bleeding (VARC-2) (median f/u of 17 months)	Higher all-cause death with rivaroxaban (HR: 1.69); numerically higher bleeding with rivaroxaban (5.6% vs. 3.8%, *p* = 0.08); trial stopped early
**ENVISAGE-TAVI AF (2021) [33]**	*n* = 1426, AF after TAVI	Edoxaban vs. VKA (INR target: 2.0–3.0)	Net adverse clinical events (death from any cause, MI, stroke, embolism, valve thrombosis, or major bleeding) at 1.5 years	Major bleeding (BARC ≥ 3): 9.7% edoxaban vs. 7% VKA (↑ GI bleeding, 5.4% vs. 2.7%)
**ATLANTIS (2021–22) [32]**	*n* = 1510, all-comers	Apixaban vs. standard of care (VKA or antiplatelet)	Composite of death, stroke, MI, embolism, intracardiac or bioprosthetic valve, and major bleeding events at 1 year	No difference in primary endpoint (*p* = 0.43); less CT-detected leaflet thrombosis with apixaban (HR: 0.23); ↑ non-CV mortality (signal)
**TRITAVI REGISTRY (2024) [30]**	*n* = 5514, multicenter	SAPT vs. DAPT	Major/life-threatening bleeding, ischemic events (death, MI, stroke), and all-cause mortality	Major bleeding: 0.5% SAPT vs. 1.3% DAPT (*p* = 0.001); ischemic events, 0.4% vs. 0.7% (*p* = 0.07) (NS); all-cause mortality lower with SAPT (2.4% vs. 5.2%, HR: 0.46, *p* = 0.005)

OAC: oral anticoagulation, SAPT: single antiplatelet therapy, DAPT: dual antiplatelet therapy, MI: myocardial infarction, HR: hazard ratio, TAVI: transcatheter aortic valve implantation, VARC: Valve Academic Research Consortium, VKA: vitamin K antagonists, CV: cardiovascular, AF: atrial fibrillation, ↑: increase.

**Table 3:** Major randomized and comparative studies on large bore transfemoral closure devices and techniques.

Study (Year)	Design and *n*	Closure Techniques Compared	Primary Endpoint	Key Outcomes
**CHOICE-CLOSURE (2022) [51]**	RCT, *n* = 516	MANTA (plug-based) vs. dual ProGlide (suture-based)	Access-site vascular complications within 30 days (VARC-2)	Device success: MANTA: 89.9% vs. Proglide × 2: 96.9%, *p* < 0.001 Primary outcome: 19.4% MANTA vs. 12.0% dual ProGlide (*p* = 0.04); life-threatening or major bleeding (BARC = 3–5) numerically higher with MANTA, 7.4% vs. 4.5% (*p* = 0.21)
**van Wiechen et al. (2021) [52]**	Pilot RCT, *n* = 210	MANTA (plug-based) vs. dual ProGlide (suture-based)	Access-site vascular complications (VARC-2)	Device success: MANTA: 98% vs. dual ProGlide: 99%, *p* = NS Vascular complications: 10% MANTA vs. 4% dual ProGlide, *p* = NS; access-site-related major bleeding complications were similar (4% vs. 4%, *p* = NS)
**ProGlide vs. Prostar XL (2020) [53]**	Observational, *n* = 2583	Dual ProGlide (suture-based) vs. Prostar XL (suture-based)	Adverse events at 30 days and 1 year (CV mortality, bleeding, and vascular complications)	Device success: 99.2% dual ProGlide vs. 97.5% Prostar (*p* = 0.001); at 30 days, lower composite primary endpoint (13.8% vs. 20.5%, *p* = 0.031) with fewer major bleeding complications, 9.1% vs. 11.7%, *p* = 0.032, with ProGlide
**ACCESS-TAVI (2025) [49]**	RCT, *n* = 400	Hybrid (ProGlide plus AngioSeal) vs. dual ProGlide	Access-site-related vascular complications at 30 days	Device success: 98.0% hybrid vs. 93.4% dual ProGlide (*p* = 0.001) Significantly fewer vascular complications and bleeding with hybrid strategy than dual ProGlide (5.9% vs. 12.8%, *p* = 0.02). Numerically fewer major and life-threatening bleeding events with hybrid approach, 1.5% vs. 4.6% (*p* = 0.09%)
**Yeh et al. (2025) [50]**	RCT, *n* = 200	Hybrid (ProGlide plus AngioSeal) vs. dual ProGlide	Hemostasis success and access-site-related vascular complications within 30 days (VARC-2)	Device success: 99% hybrid vs. 94% dual ProGlide (*p* = 0.05) Hybrid strategy reduced significant major life-threatening bleeding (BARC ≥ 3)/vascular events vs. dual ProGlide (2.0% vs. 8.7%, *p* = 0.04 and 3.9% vs. 12.6%, *p* = 0.03, respectively)

RCT: randomized controlled trial, BARC: Bleeding Academic Research Consortium, NS: not significant, VARC: Valve Academic Research Consortium, CV: cardiovascular.

## Data Availability

No new data were created or analyzed in this study.
